# Adaptation of the Behavioural Regulation in Active Commuting to School (BR-ACS) Questionnaire in Portuguese Youth

**DOI:** 10.3390/children9020182

**Published:** 2022-02-01

**Authors:** Adilson Marques, Thiago Santos, Yolanda Demetriou, Dorothea M. I. Schönbach, Miguel Peralta, Pål Lagestad, João Martins, Dorota Kleszczewska, Anna Dzielska, Élvio R. Gouveia

**Affiliations:** 1 CIPER, Faculdade de Motricidade Humana, Universidade de Lisboa, 1499-002 Lisbon, Portugal; amarques@fmh.ulisboa.pt (A.M.); mperalta@fmh.ulisboa.pt (M.P.); jmartins@fmh.ulisboa.pt (J.M.); 2 Instituto de Saúde Ambiental (ISAMB), Faculdade de Medicina, Universidade de Lisboa, 1649-028 Lisbon, Portugal; 3 Faculdade de Ciências Sociais e Tecnologia, Universidade Europeia, 1500-210 Lisbon, Portugal; thiago_os@hotmail.com; 4 Department of Sport and Health Sciences, Technical University of Munich, 80992 Munich, Germany; yolanda.demetriou@tum.de (Y.D.); dorothea.schoenbach@tum.de (D.M.I.S.); 5 Department of Arts and Teacher Education, Nord University, 7600 Levanger, Norway; pal.a.lagestad@nord.no; 6 Institute of Mother and Child Foundation, 01-211 Warsaw, Poland; dorota.kleszczewska@imid.med.pl; 7 Department of Child and Adolescent Health, Institute of Mother and Child, 01-211 Warsaw, Poland; anna.dzielska@imid.med.pl; 8 Department of Physical Education and Sport, University of Madeira, 9000-390 Funchal, Portugal; 9 LARSYS, Interactive Technologies Institute, 9020-105 Funchal, Portugal

**Keywords:** physical activity, sports, exercise, school, commute, adaptation and validation

## Abstract

This study aimed to translate and adapt the psychometric properties of the Behavioural Regulation in Active Commuting to School (BR-ACS) questionnaire to young Portuguese students. This study had two stages: (1) translation and adaptation of the questionnaire; (2) evaluation of the psychometric properties. A sample of 338 participants (212 female, 126 male) aged 11 to 19 years (M_age_ = 15.6 ± 2.1) from 31 cities and Madeira island participated in this study. The confirmatory factor analysis suggested an acceptable fit to the data for the first-order and third-order measurement models. The composite reliability values ranged from 0.71 (identified regulation) to 0.90 (integrated regulation), demonstrating internal consistency. The AVE values ranged from 0.40 (amotivation) to 0.69 (integrated regulation), demonstrating an acceptable convergent validity for all constructs. The model estimation had an acceptable fit, with values akin to those of the first-order tested model. Finally, the results of the multigroup analysis for the successive restricted models (CFI < 0.010 and RMSEA < 0.015) point out that the null hypothesis of factor invariance between gender cannot be rejected. The psychometric properties demonstrates the suitability of this questionnaire among Portuguese youths aged 11 to 19. This questionnaire will help understand the motivation aspects that underpin active commuting to school and consequently help to increase physical activity among Portuguese adolescents.

## 1. Introduction

Despite the health benefits associated with physical activity and public health recommendations [1], the physical activity level is decreasing during adolescence [2], and most young people worldwide are physically inactive [3,4]. This scenario has raised concern and led to the promotion of several strategies to enhance youth’s physical activity levels. Among those strategies, active commuting (most commonly walking or cycling) to school (ACS) is a low-cost and ecological physically active behaviour, suggested by studies to be a strategy to increase adolescents’ total physical activity [5,6,7], and thereby their health [8,9].

To promote active commuting, it is essential to recognise and understand the determinants of this behaviour. From a socio-ecologic perspective [10], these determinants can be individual, social, organisational/community, environmental and public policy [11]. The socio-ecologic model focus on the interaction of person-level attributes, such as motivation and self-efficacy, with physical and socio-cultural environments [12]. At the individual level, motivation is a key predictor of behaviour [13]. Motivation is a construct with different regulatory styles on a continuum of relative autonomy or self-determination. Intrinsic motivation is at the end of this continuum. Conversely, extrinsic motivation comprises a number of regulatory styles [14]. Having information on individual factors, especially motivation, would increase the understanding of the higher or lower engagement in this behaviour and the design of tailored strategies that effectively promote ACS among young people. Most research focused on other perspectives, such as the barriers or facilitators to physical activity, while fewer studies focus on individual approaches, such as adolescents’ own perception [15].

With that purpose in mind, Burgueño, González-Cutre [16] developed the Behavioural Regulation in Active Commuting to and from School (BR-ACS) questionnaire, based on the Behavioural Regulation in Exercise Questionnaire-3 [17]. The BR-ACS questionnaire is valid and helps to understand the motivational processes for ACS among young Spanish people [16]. Similarly to the Spanish context, the Portuguese version of the Behavioural Regulation in Exercise Questionnaire-3 already exists [18]. However, ACS is a physical activity specific to children and adolescents and concerning a specific context of practice, contributing to young people’s physical activity. Therefore, a specific instrument for assessing ACS is important.

To expand the usage of this questionnaire and obtain a greater understanding on how motivation underpins ACS behaviour across different regions worldwide, it is necessary to translate and culturally adapt the BR-ACS questionnaire to other languages and cultures. The translation and adaptation of this questionnaire are important to advance research and practical strategies for increasing ACS, especially in those contexts where young people tend to present low levels of ACS, as in Portugal [19,20]. Therefore, this study aimed to adapt the BR-ACS questionnaire to the Portuguese context and evaluate the psychometric properties of the BR-ACS questionnaire in young Portuguese students.

## 2. Materials and Methods

### 2.1. Study Design and Participants

This is a cross-sectional study developed in two different stages. The first stage was the translation and cultural adaptation of the questionnaire. The second stage was the evaluation of the questionnaire’s psychometric properties in its Portuguese version. For that purpose, students participated in a brief online questionnaire. Selected middle and high public schools were approached by directly communicating with physical education teachers. Physical education teachers invited all students to participate in the study. Students participated voluntarily and were informed about the study aims before completing the questionnaire. Before their participation, legal guardians were asked to give and sign an informed consent.

The sample comprised 338 participants (212 female and 126 male) aged 11 to 19 [mean age = 15.6± = 2.1] from 31 Portuguese cities of the mainland and Madeira island.

All participants attended public schools at the middle-school (grades 5 to 9, *n* = 166) and high-school levels (grades 10 to 12, *n* = 172). Most participants had a bicycle (63.0%) and knew how to ride a bike (83.8%). Furthermore, the great majority had a close family member who owned a car (91.9%).

Participants mainly commuted to and from school using a passive mode (42.2% by car, 37.9% by public transportation, and 0.3% by scooter). In comparison, a minor percentage used an active commuting mode (17.9% walking and 0.3% by cycling), and 1.5% used another way to commute to and from school. Accordingly, 68.5% of participants reported never walking to and from school on a single day, and only 17.6% reported walking to and from school on every day of school. The vast majority of participants, i.e., 98.8%, reported never cycling to and from school.

### 2.2. Questionnaire

The BR-ACS questionnaire consists of 23 items. These 23 items were grouped in four items per factor to measure intrinsic motivation, integrated regulation, introjected regulation, external regulation and amotivation, and three items to assess identified regulation. The items have a five-point Likert scale response option from 0 (not true) to 4 (very true). The questionnaire was validated in a sample of Spanish youth, revealing appropriate fit indices in the six-factor correlated model confirmatory factor analysis [χ2 (215, N = 404) = 550.17, *p* < 0.001, χ2/df = 2.56; CFI = 0.93; IFI = 0.93; TLI = 0.92; SRMR = 0.050; RMSEA = 0.062 (90% CI = 0.056, 0.069); BIC = 916.26] and internal consistency for intrinsic motivation (α = 0.91), integrated regulation (α = 0.90), identified regulation (α = 0.78), introjected regulation (α = 0.70), external regulation (α = 0.71), and amotivation (α = 0.70). [16].

Following the recommendations for translation and cross-cultural adaptation of questionnaires [21], two independent translators performed the first procedure (i.e., translating the BR-ACS questionnaire into an adaption to Portuguese). This procedure led to two Portuguese versions of the questionnaire. Next, the two translators compared and discussed these versions, which resulted in a first Portuguese version. Afterwards, three experts reviewed the first Portuguese version of the BR-ACS questionnaire for semantic, idiomatic, conceptual, and cultural equivalences, giving recommendations on the intelligibility of the instructions and the questionnaire items. The revision resulted in the Portuguese version 2 of the BR-ACS questionnaire. For back-translation, the Portuguese version 2 of the BR-ACS questionnaire was given to two other independent translators, who assessed whether the Portuguese version reflected the Spanish version’s content. Furthermore, the questionnaire was applied to a small sample of 6 participants (3 boys and 3 girls) to test the questionnaire’s acceptability and understanding. Participants’ feedback was taken into account, resulting in the final Portuguese version of the BR-ACS questionnaire.

The Portuguese version of the BR-ACS questionnaire was applied to 338 school-aged youths between June and August 2021, using an online link sent to their legal guardians after obtaining informed consent for the psychometric properties and validity evaluation. Participants were encouraged to answer the questionnaire based on their perceptions.

### 2.3. Statistical Analysis

The theoretical framework proposed a priori was used for self-determined motivation [22]. Two confirmatory factorial analyses were used to test the six-factor correlated structure of motivational regulation. The three-factor higher-order model is related to autonomous motivation, controlled motivation, and amotivation [13]. Because of the lack of normality (Mardia coefficient = 253.07, *p* < 0.01), the maximum likelihood method was chosen using a bootstrapping procedure with the replication of 5000 samples (based on the original sample), solving the violation of the multivariate normality. [23]. This method allowed for the estimation of the standard error and the 95% confidence interval (95% CI) for each statistical parameter. Data were analysed using AMOS 26.0 (SPSS Inc, Chicago, IL, USA). A good fit of the model is assumed through the value of the ratio of the chi-square ratio (χ2) and the degrees of freedom (df) when it is less than or equal to 3.0 (Hair et al., 2018). In addition, the reference values of the Comparative-of-The-Fit-Index (CFI), the Incremental Fit Index (IFI) and the Tucker-Lewis Index (TLI) must be greater than or equal to 0.90 [24]. Finally, the root-mean-square approximation error (RMSEA), with its 90% confidence interval (90% CI), and the standard root-mean-square residual value (SRMR) must be below the minimum or close to the cut-off point of 0.07 [25]. For the comparison between the models, the Bayesian Information Criterion (BIC) was used. It is assumed that the lowest BIC value should be the most preferable [23]. Standardised regression weights were acceptable with values above 0.50 [24]. Composite reliability values equal to or greater than 0.70 indicated a good internal consistency [24]. Values of Average Variance Extracted (AVE) equal to or greater than 0.50 indicate a good convergent validity [26]. The correlations between the factors showed adequate conceptual discrimination and, therefore, the discriminant validity with values equal to or lower than 0.85 [23].

According to the methodology described by Milfont and Fisher [27], a multigroup factor analysis was performed through the successive restricted models with the aim to a) determine whether the factorial structure of the survey was invariant across the variables and b) whether the item characteristics are comparable across manifest groups and gender [24]. The null hypothesis of factor invariance does not have to be rejected in the case of values below 0.010 for the CFI and 0.015 for the RMSEA [28].

## 3. Results

### 3.1. Assessment of the Proposed Scale

Confirmatory factor analysis for the first-order measurement model shows an acceptable fit to the data. [χ^2^(215) = 631.91 (*p* ˂ 0.01); χ^2^/df = 2.93; CFI = 0.90; IFI = 0.90; TLI = 0.90; SRMR = 0.05; RMSEA (90% CI) = 0.063 (0.058, 0.068); BIC = 984.91]. In addition, as shown in Table 1, the factor loadings ranged from 0.53 to 0.88. The composite reliability values ranged from 0.71 (identified regulation) to 0.90 (integrated regulation), demonstrating internal consistency.

The AVE values ranged from 0.40 (amotivation) to 0.69 (integrated regulation), indicating an acceptable convergent validity for all constructs. The correlation values between factors were greater than 0.82 for: intrinsic motivation and identified regulation; integrated regulation and identified regulation; and introjected regulation and external regulation. This scenario indicated problems of discriminant validity in the proposed first-order model [23]. Even so, it is necessary to consider that the model proposed by Burgueño, González-Cutre [16] considers an interaction relationship between these constructs through a higher-order three-factor model. In this sense, we decided to test the three-factor higher-order model and, through a comparison, perceive the most parsimonious model.

**Figure 1:**
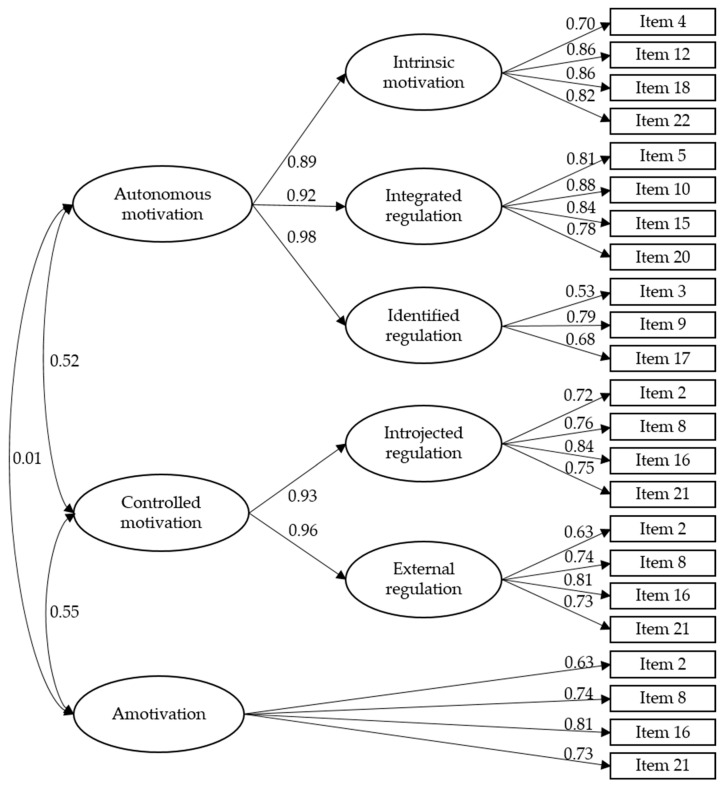
Confirmatory factor analysis.

Next, a confirmatory factor analysis was carried out for the hierarchical model of three factors composed of autonomous motivation (i.e., intrinsic motivation, integrated regulation, and identified regulation), controlled motivation (i.e., introjected and external regulation) and amotivation (see Figure 1).The fit values for the estimated model were acceptable and similar to the values of the first-order model tested. [χ^2^(222) = 652.06 (*p* ˂ 0.01); χ^2^/df = 2.93; CFI = 0.90; IFI = 0.90; TLI = 0.88; SRMR = 0.60; RMSEA (90% CI) = 0.077 (0.070, 0.084); BIC = 964.55]. The correlations among factors ranged between 0.01 and 0.55

### 3.2. Multigroup Factor Analysis of Invariance across Gender

Finally, the results of the multigroup analysis for the successive restricted models (CFI < 0.010 and RMSEA < 0.015) indicate that the null hypothesis of factor invariance between gender cannot be rejected (see Table 2).

## 4. Discussion

The aim of this study was to translate and evaluate the psychometric properties and validity of the BR-ACS questionnaire for Portuguese young people. The confirmatory factor analysis of the hierarchical three-factor model composed of autonomous motivation, controlled motivation, and amotivation showed an acceptable fit and invariance across gender. Thus, the findings support using the Portuguese version of the BR-ACS questionnaire as a valid and reliable questionnaire for behavioural (motivational) regulation on ACS among Portuguese youth.

The construct validity of the three-factor order dimension measurement model for the BR-ACS questionnaire (i.e., autonomous motivation, controlled motivation, and amotivation), presented results that are similar to those of the previous research on the same questionnaire [16]. Furthermore, in agreement with previous research, the internal consistency of the questionnaire was found to be acceptable [16]. These findings suggest the adequacy of the Portuguese version of the BR-ACS questionnaire to assess behavioural regulation incidents on motivation in the Portuguese context. These results are in line with the conceptual model analysis proposed by Burgueño et al. (2019) to assess the three-factor self-determined motivation [16].

The invariance across gender in the Portuguese version of the BR-ACS questionnaire was supported by the multigroup factor analysis, which is in accordance with the validation of the same questionnaire in Spanish young people [16]. This characteristic is important, as it specifies that the BR-ACS questionnaire can be applied to both boys and girls. Furthermore, it suggests that the questionnaire explores the possible differences concerning the motivational regulation of ACS between genders [16].

As Burgueño’s study [16], our study found a high correlation between the three autonomous and the two controlled forms of motivation. The high correlation among the forms of autonomous motivation was also founded in other studies with adolescents using another instrument to evaluate motivation in the physical education context [29]. Adolescents may have difficulty distinguishing the identified regulation (when they practise the activity because it is important) and intrinsic motivation (when they want to do the activity because it is enjoyable) [30].

The association between autonomous motivation, controlled motivation, and amotivation observed in this study, together with the validity of the three-factor dimension order model, sustains the idea of three general types of motivation, proposed by Ryan and Deci [13]. Previous research also found these dimensions to be associated with ACS [16]. These dimensions are important because they reflect motivation’s role in regulating ACS behaviour among youth, similarly to physical activity behaviour [31].

The present study has some limitations that must be acknowledged. Firstly, the convergent validity of the items in the “identified regulation” and “amotivation” dimensions was slightly below the reference value (AVE = 0.50). Some factor loadings of items in both dimensions presented values close to the minimum reference value of 0.50, which may have penalised the convergent validity values. This may be related to the lack of clarity of the questions. A refinement of the items is proposed in future studies, as it could favour a better understanding on the part of respondents and a better saturation of the item in the factor. Secondly, this was a cross-sectional study, and thus it is not possible to assess the associations’ direction. This means that it is not possible to say whether the motivation preceded the ACS behaviour, or if the ACS behaviour preceded the motivation. Finally, the specific Portuguese context where the BR-ACS questionnaire was applied precludes a generalisation of the results. Moreover, the sample has not been randomly selected in all regions of Portugal, so a generalisation to the entire country is not possible. In addition, we did not consider the distance between school and the students’ home in the analysis. Future studies focusing on other contexts and languages should adapt this questionnaire linguistically and culturally to confirm its validity and randomly select participants.

## 5. Conclusions

The translation and validation of the BR-ACS Portuguese version questionnaire, adapted from the BR-ACS questionnaire’s Spanish version [16], are presented in this study. The Portuguese version of the BR-ACS questionnaire is valid for the Portuguese context. The assessment of psychometric properties provided evidence of the suitability of this questionnaire among Portuguese youths aged 11 to 19. With this instrument, the researchers could acquire a better understanding of the motivational aspects regarding active adolescents commuting to and from school and make decisions to promote a more active transportation and increase physical activity, thereby improving adolescents’ health.

## Figures and Tables

**Table 1:** Means and Standard Deviations (M, SD); Factor loading; construct reliability (CR); average variance extracted (AVE); and correlations among constructs.

Constructs/Items	M (SD)		Loadings		CR	AVE
*Intrinsic Motivation*					0.89	0.67
4. Vou para a escola a pé ou de bicicleta porque é divertido. (I walk or cycle to and from school because it is fun)	1.15 (1.41)		0.70			
12. É agradável ir para a escola a pé ou de bicicleta. (I enjoy walking or cycling to and from school)	1.79 (1.55)		0.86			
18. Acho que ir para a escola a pé ou de bicicleta é agradável. (I find walking or cycling to and from school a pleasurable activity)	1.89 (1.54)		0.86			
22. Gosto de ir para a escola a pé ou de bicicleta. (I like walking or cycling to and from school)	1.52 (1.53)		0.82			
*Integrated Regulation*					0.90	0.69
5. Vou para a escola a pé ou de bicicleta porque isso está de acordo com a minha maneira de ser. (I walk or cycle to and from school because it is consistent with my life goals)	1.10 (1.46)		0.81			
10. Considero que ir para a escola a pé ou de bicicleta faz parte da minha identidade. (I consider walking or cycling to and from school to be part of my identity)	0.84 (1.32)		0.88			
15. Sinto que ir para a escola a pé ou de bicicleta é uma parte fundamental de quem eu sou. (I consider walking or cycling to and from school a fundamental part of who I am)	0.90 (1.33)		0.84			
20. Considero que ir para a escola a pé ou de bicicleta está de acordo com meus valores. (I consider walking or cycling to and from school are consistent with my values)	1.23 (1.48)		0.78			
*Identified Regulation*					0.71	0.46
3. Dou valor aos benefícios/vantagens de ir a pé ou de bicicleta para a escola. (I value the benefits of walking or cycling to and from school)	2.54 (1.43)		0.53			
9. É importante para mim ir para a escola a pé ou de bicicleta. (It is important to me to walk or cycle to and from school regularly)	1.16 (1.45)		0.79			
17. Penso que é importante fazer um esforço por ir para a escola a pé ou de bicicleta regularmente. (It is important to make an effort to walk or cycle to and from school regularly)	1.62 (1.55)		0.68			
*Introjected Regulation*					0.86	0.60
2. Sinto-me culpado quando não vou para a escola a pé ou de bicicleta. (I feel guilty when I do not walk or cycle to and from school)	0.43 (0.914)		0.72			
8. Sinto-me envergonhado quando não vou para a escola a pé ou de bicicleta. (I feel ashamed when I do not walk or cycle to and from school)	0.27 (0.76)		0.76			
16. Sinto-me fracassado quando não vou para a escola a pé ou de bicicleta. (I feel a failure when I have not walked or cycled to and from school)	0.30 (0.84)		0.84			
21. Sinto-me ansioso se não for para a escola a pé ou de bicicleta. (I get restless if I do not walk or cycle to and from school regularly)	0.37 (0.94)		0.75			
*External Regulation*					0.82	0.54
1. Vou a pé ou de bicicleta para a escola porque outras pessoas dizem que o devo fazer. (Because other people say I should walk or cycle to and from school)	0.37 (0.90)		0.63			
7. Vou para a escola a pé ou de bicicleta porque os meus amigos/família/professores dizem que o devo fazer. (Because my friends/family/teacher say I should walk or cycle to and from school)	0.44 (0.95)		0.74			
13. Vou para a escola a pé ou de bicicleta porque os outros vão ficar insatisfeitos comigo se o não fizer. (Because others will not be pleased with me if I do not walk or cycle to and from school)	0.27 (0.82)		0.81			
19. Sinto-me pressionado pela minha família e amigos para ir para a escola a pé ou de bicicleta. (I feel pressured by my friends/family to walk or cycle to and from school)	0.37 (0.97)		0.73			
*Amotivation*					0.72	0.40
6. Não vejo porque é que tenho de ir para a escola a pé ou de bicicleta. (I do not see why I should have to walk or cycle to and from school)	1.13 (1.38)		0.61			
11. Não percebo porque me devo preocupar em ir para a escola a pé ou de bicicleta. (I cannot see why I should bother walking or cycling to and from school)	1.22 (1.49)		0.61			
14. Não percebo o objetivo de ir para a escola a pé ou de bicicleta. (I do not see the point in walking or cycling to and from school)	0.83 (1.26)		0.73			
23. Penso que ir para a escola a pé ou de bicicleta é uma perda de tempo. (I think that walking or cycling to and from school is a waste of time)	0.74 (1.14)		0.54			
Constructs/Correlations	**1**	**2**	**3**	**4**	**5**	**6**
1. Intrinsic Motivation	1.00					
2. Integrated Regulation	0.82	1.00				
3. Identified Regulation	0.90	0.89	1.00			
4. Introjected Regulation	0.37	0.52	0.50	1.00		
5. External Regulation	0.37	0.53	0.45	0.90	1.00	
6. Amotivation	−0.03	0.05	−0.01	0.49	0.54	1.00

**Table 2:** Multigroup factor analysis of invariance.

	X^2^	df	X^2^/df	CFI	IFI	TLI	SRMR	RMSEA (90%CI)	MC	∆X^2^	∆df	∆CFI	∆RMSEA
Configural invariance	969.62	430	2.25	0.87	0.87	0.85	0.062	0.063 (0.058, 0.068)	--	--	--	--	--
Metric invariance	1012.98	447	2.26	0.87	0.87	0.85	0.063	0.063 (0.058, 0.068)	2 vs. 1	43.36 *	17	0.000	0.000
Scalar invariance	1023.45	464	2.20	0.87	0.87	0.86	0.064	0.062 (0.056, 0.067)	3 vs. 2	10.47	17	0.000	-0.001
Error variance invariance	1106.58	487	2.27	0.86	0.86	0.85	0.065	0.063 (0.058, 0.068)	4 vs. 3	83.13 *	23	0.002	0.000

Notes: *p < 0.01.

## Data Availability

The datasets generated and/or analysed during the current study are not publicly available due to the terms of consent/assent to which the participants agreed but are available from the corresponding author upon reasonable request. Please contact the corresponding author to discuss the availability of the data and materials.
